# Two closely related species differ in their regional genetic differentiation despite admixing

**DOI:** 10.1093/aobpla/ply007

**Published:** 2018-01-29

**Authors:** Lisanna Schmidt, Markus Fischer, Tatjana Oja

**Affiliations:** 1 Institute of Ecology and Earth Sciences, Department of Botany, University of Tartu, Tartu, Estonia; 2 Institute of Plant Sciences, University of Bern, Bern, Switzerland; 3 Botanical Garden, University of Bern, Bern, Switzerland; 4 Oeschger Centre for Climate Change Research, University of Bern, Bern, Switzerland

**Keywords:** *Carex flava* complex, genetic diversity, hybridization, microsatellites, population differentiation

## Abstract

Regional genetic differentiation within species is often addressed in evolutionary ecology and conservation biology. Here, we address regional differentiation in two closely related hybridizing taxa, the perennial sedges *Carex flava* and *C. viridula* and their hybrid *C*. × *subviridula* in 37 populations in the north and centre of their distribution range in Europe (Estonia, Lowland (<1000 m a.s.l.) and Highland Switzerland) using 10 putative microsatellite loci. We ask whether regional differentiation was larger in the less common taxon *C. viridula* or whether, possibly due to hybridization, it was similar between taxa. Our results showed similar, low to moderate genetic diversity for the three studied taxa. In total, we found 12 regional species-specific alleles. Analysis of molecular variance (AMOVA), STRUCTURE and multidimensional scaling analysis showed regional structure in genetic variation, where intraspecific differentiation between regions was lower for *C. flava* (AMOVA: 6.84 %) than for *C. viridula* (20.77 %) or *C.* × *subviridula* (18.27 %) populations. Hybrids differed from the parental taxa in the two regions where they occurred, i.e. in Estonia and Lowland Switzerland. We conclude that *C. flava* and *C. viridula* clearly differ from each other genetically, that there is pronounced regional differentiation and that, despite hybridization, this regional differentiation is more pronounced in the less common taxon, *C. viridula*. We encourage future studies on hybridizing taxa to work with plant populations from more than one region.

## Teaser

Genetic differences between regions are usually studied for individual species. However, many species can reproduce with each other. We studied whether gene flow between two closely related sedge species influences regional differences. Our molecular genetic data support considerable gene flow between the species. Still, we detected clear genetic differences between species and regions, and more pronounced regional differences for the less common one. Thus, gene flow between the species appeared too weak to neutralize differences between the regional genetic structure of our study species. We encourage further regional differentiation studies in groups of cross-compatible species.

## Introduction

Plants and other organisms differ in their levels of genetic diversity and genetic differentiation (Linhart and Grant 1996). The extent of genetic differentiation among populations and regions depends on the balance of evolutionary forces decreasing and increasing genetic differentiation, that is, gene flow, genetic drift, mutation and selection (Slatkin 1987). The relative importance of these forces may be affected by selection strength, as well as population size, environmental barriers to dispersal and plant life history traits, especially mating system and dispersal mechanism (Loveless and Hamrick 1984). Higher differentiation among populations is generally found for clonally reproducing and selfing species (Loveless and Hamrick 1984; Hamrick and Godt 1996; Nybom 2004; Song *et al.* 2006) and for species with disjunct distributions or small populations (Ellstrand and Elam 1993), due to effects of genetic drift or reduced gene flow (Slatkin 1987; Schönswetter *et al.* 2006). Outcrossing and sexually reproducing species, conversely, show less differentiation among populations (Hamrick *et al.* 1979; Loveless and Hamrick 1984).

While genetic consequences of small population size, i.e. increased inbreeding and genetic drift, are expected to contribute to higher population differentiation and lower genetic diversity within populations (Slatkin 1987; Ellstrand and Elam 1993; Amos and Harwood 1998; Premoli 2003; Leimu *et al.* 2006; Zhivotovsky *et al.* 2016), this is not found in all studies (Gitzendanner and Soltis 2000). Loveless and Hamrick (1984) agree that small populations are more susceptible to drift and fixation, but suggest that immigration is more effective in altering gene frequencies in small populations. Thus, gene flow can prevent differentiation and loss of genetic variability, especially in long-lived species. Levels of genetic diversity in populations may also depend on distance from glacial refugia (Schönswetter *et al.* 2006) and on the landscape (Holderegger and Wagner 2006). For populations at higher altitudes higher radiation intensity may increase the rate of mutations and thus population genetic diversity (Li *et al.* 1997).

It is generally assumed that species are reproductively isolated without gene flow between them, but in reality hybridization is a widespread phenomenon (Ellstrand *et al.* 1996; Abbott *et al.* 2013) and becomes more frequent with continuous climate change and human influence on the environment (Hoffmann and Sgro 2011). Gene flow between taxa can have a profound effect on genetic diversity (Derieg *et al.* 2008), and due to subsequent genetic drift, may lead to population differentiation (Bain and Golden 2003). Hybridization of closely related species is found to lead to genetic differentiation among regions (Rieseberg *et al.* 1999; Kane *et al.* 2009; Lepais *et al.* 2009; Krebs *et al.* 2010; Brennan *et al.* 2016). According to the ‘semipermeable species boundaries’ theory, alleles at some loci can be exchanged between species and species boundaries can vary geographically (Harrison and Larson 2014). Thus, hybridization coupled with backcrossing can be expected to affect population differentiation on a regional scale.

Hybrid individuals are expected to be more heterozygous than their parental taxa due to genetic admixing (Rieseberg and Wendel 1993; Allendorf *et al.* 2001; Barton 2001; Harrison and Larson 2014; Todesco *et al.* 2016). Hybridization could result in beneficial evolution by providing additional adaptive genetic variation (Lewontin and Birch 1966; Arnold 2006; Abbott *et al.* 2013). Conversely, hybridization can reduce biodiversity by causing loss of alleles and genetic diversity by genetic assimilation (Levin *et al.* 1996). Genetic studies on hybridizing taxa have focused on their genetic variation or the extent of hybridization and introgression (e.g. Friedman *et al.* 2008; Volkova *et al.* 2008; Kane *et al.* 2009; Korpelainen *et al.* 2010; Krebs *et al.* 2010). Our study addresses the differentiation of hybrids from parental taxa.

We investigate genetic diversity and regional differentiation between and within *Carex flava*, *C. viridula* and their hybrid *C.* × *subviridula* for populations from three regions (Estonia in Northern Europe and Lowland and Highland Switzerland in Central Europe). *Carex flava* and *C. viridula* var. *viridula sensu stricto* (*s.s.*) (henceforth *C. viridula*) are wind-pollinated, self-compatible, caespitose perennials of the *C. flava* aggregate (*Carex* sect. *Ceratocystis*, Cyperaceae). Although there are no impediments to outcrossing, a large amount of seeds is produced by selfing (Vonk 1979; Schmid 1984a). Both taxa, with circumpolar distribution, occur in the temperate and subarctic Northern hemisphere and also in North Africa (Hultén and Fries 1986; Crins and Ball 1989; Koopman 2011). They often co-occur and hybridize, especially at sites with disturbances (Vonk 1979; Jiménez-Mejías *et al.* 2012), resulting in *C.* × *subviridula*. *Carex viridula* is considered to be a dispersal generalist, potentially being transported by biotic, e.g. birds, mammals, invertebrates, and abiotic agents, e.g. water and wind (Schmid 1984a; Crins and Ball 1989). Long-distance dispersal is proven experimentally with *C. flava* var. *alpina* seeds remaining still partly intact after passing the digestive tract of domesticated ducks within 18 h (Schmid 1984a). *Carex viridula* is a weak competitor, but able to colonize and survive in fluctuating, relatively unpredictable moist or wet habitats, where it forms small populations (Schmid 1986; Crins and Ball 1989; Kuchel and Bruederle 2000). However, populations of *C. viridula* are sensitive to anthropogenic influence, such as the drainage of mires, regulation of water levels and eutrophication of shores (Pykälä and Toivonen 1994) and its decrease in southern parts of its distribution could be explained by climate change (Parmesan and Yohe 2003; Chen *et al.* 2011). Hence, the occurrence of *C. viridula* has decreased over time and populations have become more fragmented (Davies 1953; Pykälä and Toivonen 1994). According to the latest red list of endangered plants in Switzerland, it is considered near threatened due to loss of habitat (Bornand *et al.* 2016). *Carex flava*, on the other hand, is a strong competitor and not as sensitive to environmental changes; its populations are larger and rather constant in time (Schmid 1984a, b, 1986). Intraspecific gene flow is expected to be higher for *C. flava* and smaller for *C. viridula* (Schmid 1986).

Previous genetic studies with the *C. flava* agg. were restricted to small regions, did not consider co-occurrence and hybridization or used allozyme markers of limited variability (Bruederle and Jensen 1991; Kuchel and Bruederle 2000; Hedrén 2002, 2004; Blackstock and Ashton 2010; except Jiménez-Mejías *et al.* 2012). The novelty of our study originates from considering co-occurrence and hybridization, from comparing geographically and climatically distant regions, and from using contemporary molecular microsatellite (SSR) markers. According to our hypothesis, admixture, i.e. hybridization coupled with backcrossing, can be expected to affect population differentiation on a regional scale. Therefore, species characterized by relatively small populations might be less well differentiated on a regional level than expected without admixing. Thus, we study whether, despite admixing, *C. viridula*, characterized by relatively small populations, exhibits higher levels of population differentiation than *C. flava*, which has relatively large populations. In addition, we investigate the variability of hybrid populations and their differentiation from the parental taxa.

## Methods

### Study taxa and regions

In the studied regions, the *C. flava* group comprises the four taxa *C. flava*, *C. lepidocarpa*, *C. demissa* and *C. viridula* var. *viridula* (Schmid 1981; Toom *et al.* 2016). In Estonia, the two varieties *C. viridula* var. *pulchella* and var. *bergrothii* are also found (Toom *et al.* 2016). In this study, we follow Hedrén (2002, 2004) taxonomic treatment, but we use the more common name *C. viridula* instead of calling it *C. oederi*.

The studied regions differ in their postglacial history. Swiss populations were established earlier after glaciation, because the territory of Estonia was covered by ice at the end of the last ice age 18000 BP, when the lower parts of Switzerland were ice-free (Hewitt 1999). In addition, the current populations in Switzerland are much closer to possible southern glacial refugia in Iberia, the Apennine or the Balkan Peninsula (Schönswetter *et al.* 2006) than Estonian populations are. As the populations in Switzerland are older, they may be more amalgamated via interspecific gene flow, hybridization and introgression than the Estonian ones.

### Population sampling

**Figure 1.**
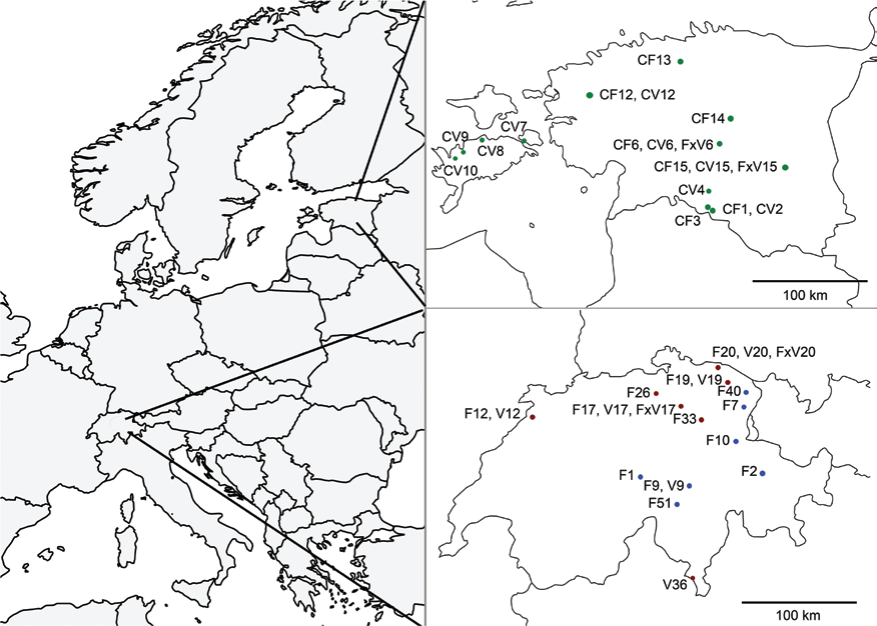
Sampling sites in Switzerland and Estonia (top right). For Switzerland blue dots indicate Highland Switzerland populations, dark red dots Lowland Switzerland populations. Sites, where more than one taxon was co-occurring, are marked with one dot. Population codes can be found in Table 1.

We collected 380 samples of *C. flava*, *C. viridula* and *C.* × *subviridula* populations from seven sites in Highland Switzerland (>1000 m, in 2012), five sites in Lowland Switzerland (<1000 m, in 2012) and 12 sites in Estonia (2013; Table 1; Fig. 1). At 15 sites we found *C. flava* and *C. viridula* growing together or with other sedges, with whom they are able to hybridize, i.e. with other *C. flava* aggregate members or *C. punctata* and *C. hostiana* (Davies 1955; Schmid 1982; Crins and Ball 1989; Więcław and Wilhelm 2014). Samples of the *C. flava* × *C. viridula* hybrid (*C.* × *subviridula*) were found at two sites in Estonia and two in Lowland Switzerland, while none were found in Highland Switzerland. As hybrids, we classified partly sterile individuals that were morphologically intermediate between the parents, but often were more robust and pale (Schmidt *et al.* 2017). The sampling populations of *C. viridula* were smaller, i.e. had fewer individuals, than the ones of *C. flava*. As we focus on between-region and between-taxa comparisons, for which the population is the unit of replication against which between-region and between-taxa differences are tested, and as the power of such analyses depends on the number of populations, whereas the replication within populations is less decisive (van Kleunen *et al.* 2014), we sampled a total of 37 populations and on average 10.3 individuals per population.

**Table 1.** Populations (Pop) and geographic locations of *Carex flava* (sect. *Ceratocystis*, Cyperaceae), *Carex viridula* var. *viridula* and their hybrid (*C.* × *subviridula*) from three regions: Estonia (EST), Highland (CHH) and Lowland Switzerland (CHL). *n*, number of analysed individuals; m, altitude in m a.s.l.; N, latitude; E, longitude. The sites where *C. flava* and *C. viridula* grew together with other members of the *C. flava* agg. are termed ‘mixed’, and the sites where only *C. flava* or only *C. viridula* occurred ‘pure’. Note: population name codes sharing the same number indicate populations co-occurrence at the same site.

Species (*n*)	Pop	State	Location	m	N	E
Highland Switzerland (CHH)						
*C. flava* (12)	F2	Mixed	Arosa, Peist	1920	46.8013	9.6841
*C. flava* (6)	F1	Pure	Melchsee-Frutt, Kerns	1910	46.7704	8.2809
*C. flava* (18)	F7	Mixed	Rüte	1158	47.3221	9.4695
*C. flava* (6)	F9					
*C. viridula* (8)	V9	Mixed	Fontanivas, Disentis	1046	46.6989	8.8565
*C. flava* (14)	F10	Mixed	Chapfensee, Mels	1030	47.0483	9.3770
*C. flava* (7)	F12					
*C. viridula* (4)	V12	Mixed	Etang de Gruere, Saignelegier	1007	47.2381	7.0508
*C. flava* (8)	F40	Mixed	Gupfloch, Rehetobel	1015	47.4346	9.4988
Lowland Switzerland (CHL)						
*C. flava* (14)	F17					
*C. viridula* (11)	V17					
Hybrid (3)	FxV17	Mixed	Robenhuserriet, Wetzikon	535	47.3399	8.7811
*C. flava* (7)	F19					
*C. viridula* (14)	V19	Mixed	Hudelmoos, Amriswil	525	47.5238	9.2869
*C. flava* (32)	F20					
*C. viridula* (11)	V20					
Hybrid (12)	FxV20	Mixed	Neuweiher, Kreuzlingen	500	47.6311	9.1743
*C. flava* (6)	F33	Pure	Kaltbrunner Riet, Kaltbrunn	410	47.2152	8.9894
*C. viridula* (20)	V36	Pure	Luganersee, Caslano	270	45.9613	8.8872
Estonia (EST)						
*C. viridula* (9)	CV4	Pure	Tarvastu, Veisjärv	97	58.1060	25.7639
*C. flava* (9)	CF3	Pure	Helme, Holdre	93	57.9639	25.7434
*C. flava* (4)	CF1					
*C. viridula* (4)	CV2	Mixed	Helme, Lagesoo	87	57.9494	25.8066
*C. flava* (9)	CF14	Pure	Pajusi, Endla	86	58.7603	26.1310
*C. flava* (8)	CF13	Pure	Anija, Padriku	64	59.2959	25.3726
*C. flava* (14)	CF12					
*C. viridula* (11)	CV12	Mixed	Risti, Marimetsa bog	45	58.9932	24.0743
*C. flava* (9)	CF6					
*C. viridula* (10)	CV6					
Hybrid (10)	FxCV6	Mixed	Kolga-Jaani, Leie	37	58.4139	26.0396
*C. flava* (13)	CF15					
*C. viridula* (10)	CV15					
Hybrid (7)	FxCV15	Mixed	Luunja, Kabina	33	58.3437	26.8252
*C. viridula* (10)	CV8	Pure	Leisi, Meiuste	27	58.5860	22.5681
*C. viridula* (10)	CV9	Pure	Mustjala, Võhma	19	58.5206	22.3334
*C. viridula* (10)	CV10	Pure	Mustjala, Paatsa	6	58.5053	22.3126
*C. viridula* (10)	CV7	Pure	Muhu, Nautse	3	58.5774	23.1658

As population genetic diversity is expected to be high in the centre and to decline at the margins of distribution ranges (Volis *et al.* 2016), we studied the taxa neither in the centre nor the margins of theirs. Our southernmost population was at the latitude of 45.96 N in Caslano, Switzerland (V36), and the northernmost one at 59.29 N in Anija, Estonia (CF13). In Switzerland, the population at lowest altitude was in Caslano at 270 m a.s.l. and the one at highest altitude in Arosa at 1920 m a.s.l. Vouchers with samples of all study populations were deposited in the herbarium of the Natural History Museum of the University of Tartu.

### Microsatellite analysis

The microsatellite loci tested in this study were developed for other species. Due to good success in cross-amplification among congeners (Rossetto *et al.* 1999), we chose primer pairs isolated from other *Carex* species. We chose in total 17 polymorphic microsatellite loci from previous studies that had showed successful cross-species amplification. We screened nine primer pairs developed for *Carex scoparia* (Hipp *et al.* 2009), two primers developed for *Carex rugulosa* (Ohbayashi *et al.* 2008), four primers developed for *Carex kobomugi* (Ohsako and Yamane 2007) and two primers developed for *Carex limosa* (Escudero *et al.* 2010). Primary microsatellite analysis was performed with few individuals of each taxon and 17 primer pairs. Of the 17 primer pairs tested, 10 aligned successfully with recipient DNA, cross-amplified in *C. flava*, *C. viridula* and *C.* × *subviridula*, exhibited polymorphism and showed identifiable peaks in fragment analysis. Those 10 primer pairs were used for further analysis with all 380 samples **[see Supporting Information—Table S1]**. We did not sequence the fragments recovered for the 10 loci nor did we perform progeny analysis. However, the primer pairs used in our study had been successfully used in other population genetic studies (Hipp *et al.* 2009; Escudero *et al.* 2010; Korpelainen *et al.* 2010).

Each primer was optimized for a range of temperatures (*T*_a_: 49–60.1 °C). Magnesium source 1.2 mM MgSO_4_ was used, except for S177, where 1.6 mM MgCl_2_ was used. PCR amplifications were performed in 10 µL volumes containing 1–2 µL of genomic DNA, 1.2 µL GoTaq Flexi buffer (1×), 0.6 µL of each dNTP, 0.5 µL of untagged primer, 0.5 µL of fluorescent tag, 0.5 µL of the tagged primer, 0.05 µL of bovine serum albumin (BSA), 0.05 µL GoTaq Flexi DNA polymerase and varying concentrations of MgCl_2_ or MgSO_4_. Total genomic DNA was isolated from silica-dried leaves using the CTAB method (Doyle 1987). The extracted DNA was dissolved in 100 µL of TE buffer and diluted to 1:10 for further PCR analyses. DNA from each sample was amplified with the common tag containing one of four fluorescent dyes, 6-FAM, PET, VIC or NED (Applied Biosystems). PCRs were carried out as follows: preliminary denaturation at 95 °C for 5 min, 35 cycles at 95 °C for 1 min, annealing temperature 53.6–60 °C for 1 min, 72 °C for 1 min and a final extension step at 72 °C for 30 min, using a Techne TC-5000 thermocycler (Bibby Scientific). PCR products of different primers, each of 1–2 µL, were mixed together yielding a total of 20-µL mixture. From each mixture 2 µL were pooled with 10 µL buffer (size standard: deionized formamide = 1:25) in the wells of a 96-well plate for fragment analysis on a 3730xl DNA Analyzer (Applied Biosystems), where samples of different plant individuals were randomized. Product sizes were determined using the Peak Scanner Software v1.0 (Applied Biosystems). Scoring errors, e.g. null alleles, were identified and corrected using micro-checker 2.2.3 (van Oosterhout *et al.* 2004).

### Data analysis

For each population the effective number of alleles (*N*_e_), percentage of polymorphic loci (PL), expected heterozygosity (*H*_e_, also called gene diversity) and observed heterozygosity (*H*_o_) were estimated across all loci using GENALEX 6.5 (Peakall and Smouse 2006). Allelic richness (Ar) and compliance with Hardy–Weinberg expectations were calculated in FSTAT v 2.9.3 (Goudet 2002). The inbreeding coefficient (*F*_IS_) shows the probability to observe alleles of an individual (I) that are identical by descent (IBD) in a subpopulation (S). *F*_IS_, calculated as (*H*_e_ − *H*_o_)/*H*_e_, allowed to estimate the prevailing mating systems by region (i.e. Estonia, Lowland and Highland Switzerland) and taxon. Variation in these measures of population diversity was tested with ANOVA, using taxon, region of origin and the interaction of taxon and region of origin as fixed effects, implemented in the software R v 3.1.2 (R Development Core Team 2016).

#### Population genetic structure and hybrid identification.

We used a Bayesian clustering approach as implemented in STRUCTURE v 2.3.4 (Pritchard *et al.* 2000) (i) to estimate the number of genetic clusters (*K*) without *a priori* knowledge of taxonomy or population, and (ii) to identify the hybrid individuals with admixture analysis. The clustering was conducted with the admixture model and the correlated-allele-frequencies option using a burn-in of 10000 steps and 100000 replications, the remaining parameters were set to the default values. Five independent runs were done for the set of *K* = {1:10}. We used Structure Harvester v 0.6.92 (Earl 2012) to visualize the optimal number of clusters (*K*) by using firstly the Δ*K* method of Evanno *et al.* (2005) and secondly by examining the distribution of the log-likelihoods for the value with the highest probability and lowest variance using Markov chain Monte Carlo simulations. With *K* = 2 we detected the posterior probability (*q*-values), which describes the proportion of an individual genotype originating from each of *K* categories. We used *K* = 2 as we expected two taxa contributing to the gene pool of hybrids. We chose a threshold value of 0.9, which was found efficient to distinguish pure individuals (*q* > 0.9 or *q* < 0.1) from hybrids and backcrosses (0.1 < *q* < 0.9) (Vähä and Primmer 2006; Burgarella *et al.* 2009).

#### Genetic differentiation.

To compare the degree of differentiation among groups of populations categorized by taxa and region of origin, between-group *F*_ST_ values were calculated, using Arlequin v 3.5.2.2 (Excoffier *et al.* 2005). The significance of differences in the resulting values was tested with 1000 permutations. To illustrate the dissimilarities among groups of populations categorized by taxa and by region of origin, multidimensional scaling (MDS) analysis was performed in software R v 3.1.2 (R Development Core Team 2016) based on Reynolds distances obtained with the software Arlequin, which estimates the co-ancestry of different samples (Reynolds *et al.* 1983).

Analysis of molecular variance (AMOVA) enabled us to determine the distribution of microsatellite variation among groups of populations, among populations within groups and among individuals within populations, using Arlequin. We grouped the populations according to the tested hypotheses per taxa and regions. We tested the significance of the variance components by calculating their probabilities based on 9999 permutations of individual samples.

## Results

The total number of alleles observed per locus in the overall sample of 380 individuals from 37 populations and 24 sites of three regions ranged from 4 to 11, with overall 64 alleles scored over the 10 loci. Private alleles were detected at seven loci, four of which were found in *C. flava* in Highland Switzerland **[see Supporting Information—Table S2]**.

Total genetic diversity varied little between the three studied taxa, and the overall absolute values of genetic diversity statistics, which comprise variation within and between regions, were similar in the more common *C. flava* (*N*_e_ = 1.42, PL = 56.11 %, Ar = 1.56, *H*_e_ = 0.21) than in the less common *C. viridula* (*N*_e_ = 1.54, PL = 54.0 %, Ar = 1.64, *H*_e_ = 0.25) (Table 2). The percentage of polymorphic loci was significantly different between two regions (*F* = 10.2, *P* = 0.01) and among three regions (*F* = 5.85, *P* = 0.01; Table 3). Significant taxon-by-region interactions for allelic richness (*F* = 3.58, *P* = 0.04), percentage of polymorphic loci (*F* = 5.30, *P* = 0.01) and expected heterozygosity (*F* = 3.89, *P* = 0.03) indicate that differences between the two taxa in their levels of genetic diversity depended on the region (Table 3a).

**Table 2.** Genetic diversity of (a) *Carex flava* (sect. *Ceratocystis*, Cyperaceae), (b) *C. viridula* var. *viridula* and (c) hybrid *C.* × *subviridula* by region of origin. *n*, sample size; *N*_e_, effective number of alleles; Ar, allelic richness; PL %, percentage of polymorphic loci; *H*_o_, observed heterozygosity; *H*_e_, expected heterozygosity (gene diversity); *F*_IS_, inbreeding coefficient. For population codes follow Table 1.

(a) *C. flava*								
Region	Pop	*n*	*N*_e_	Ar	PL %	*H*_o_	*H*_e_	*F*_IS_
Overall means			**1.42**	**1.56**	**56.11**	**0.16**	**0.21**	**0.18**
CHH	F1	6	1.40	1.60	70.0	0.27	0.24	−0.12
	F10	14	1.52	1.71	80.0	0.16	0.29	0.43
	F12	7	1.28	1.34	40.0	0.19	0.15	−0.21
	F2	12	1.52	1.76	70.0	0.23	0.28	0.18
	F40	8	1.33	1.54	70.0	0.16	0.21	0.23
	F7	18	1.54	1.79	90.0	0.21	0.30	0.32
	F9	6	1.75	1.92	80.0	0.17	0.38	0.56
		Mean	**1.48**	**1.67**	**71.4**	**0.20**	**0.26**	**0.20**
CHL	F17	14	1.32	1.40	50.0	0.16	0.15	−0.04
	F19	7	1.18	1.20	20.0	0.17	0.10	−0.79
	F20	32	1.43	1.71	100.0	0.12	0.27	0.54
	F33	6	1.70	1.89	80.0	0.18	0.35	0.47
		Mean	**1.41**	**1.55**	**62.5**	**0.16**	**0.22**	**0.05**
EST	CF1	4	1.31	1.44	40.0	0.15	0.15	−0.02
	CF12	14	1.54	1.56	40.0	0.18	0.22	0.20
	CF13	8	1.10	1.16	20.0	0.06	0.06	0.04
	CF14	9	1.23	1.35	40.0	0.17	0.14	−0.18
	CF15	13	1.26	1.37	40.0	0.09	0.14	0.33
	CF3	9	1.71	1.71	40.0	0.09	0.24	0.63
	CF6	9	1.44	1.54	40.0	0.07	0.19	0.65
		Mean	**1.37**	**1.45**	**37.1**	**0.12**	**0.16**	**0.24**
(b) *C. viridula*								
Region	Pop	*n*	*N*_e_	Ar	PL %	*H*_o_	*H*_e_	*F*_IS_
Overall means			**1.54**	**1.64**	**54.0**	**0.23**	**0.25**	**0.05**
CHH	V12	4	1.24	1.30	30.0	0.15	0.13	−0.14
	V9	8	1.43	1.49	40.0	0.20	0.19	−0.04
		Mean	**1.33**	**1.40**	**35.0**	**0.18**	**0.16**	−**0.09**
CHL	V17	11	1.99	2.03	70.0	0.22	0.39	0.44
	V19	14	1.51	1.67	60.0	0.18	0.24	0.25
	V20	11	2.14	2.17	90.0	0.37	0.43	0.14
	V36	20	1.44	1.58	60.0	0.19	0.24	0.20
		Mean	**1.77**	**1.86**	**70.0**	**0.24**	**0.32**	**0.26**
(b) *C. viridula*								
Region	Pop	*n*	*N*_e_	Ar	PL %	*H*_o_	*H*_e_	*F*_IS_
EST	CV10	10	1.42	1.53	50.0	0.17	0.21	0.20
	CV12	11	1.45	1.50	40.0	0.24	0.21	−0.12
	CV15	10	1.52	1.60	60.0	0.32	0.26	−0.23
	CV2	4	1.18	1.27	30.0	0.15	0.11	−0.41
	CV4	9	1.52	1.63	40.0	0.12	0.22	0.44
	CV6	10	1.52	1.64	60.0	0.10	0.24	0.59
	CV7	10	1.43	1.51	50.0	0.26	0.21	−0.27
	CV8	10	1.61	1.82	70.0	0.28	0.30	0.05
	CV9	10	1.79	1.80	60.0	0.46	0.33	−0.40
		Mean	**1.49**	**1.59**	**51.1**	**0.23**	**0.23**	−**0.02**
(c) *C.* × *subviridula*								
Region	Pop	*n*	*N*_e_	Ar	PL %	*H*_o_	*H*_e_	*F*_IS_
Overall means			**1.52**	**1.68**	**57.0**	**0.27**	**0.24**	−**0.09**
CHL	FxV17	3	1.64	1.70	50.0	0.30	0.27	−0.10
	FxV20	12	1.34	1.65	80.0	0.21	0.22	0.07
		Mean	**1.49**	**1.68**	**65.0**	**0.25**	**0.25**	−**0.02**
EST	FxCV15	7	1.55	1.67	40.0	0.29	0.23	−0.26
	FxCV6	10	1.61	1.71	50.0	0.28	0.25	−0.13
		Mean	**1.58**	**1.69**	**45.0**	**0.28**	**0.24**	−**0.20**

Overall means per taxon and mean values per taxon and region of origin are in bold.

**Table 3.** Summary of ANOVAs testing the effects of taxon, region of origin and the interaction between taxon and region of origin on genetic diversity statistics. *N*_e_, effective number of alleles; Ar, allelic richness; PL %, percentage of polymorphic loci; *H*_o_, observed heterozygosity; *H*_e_, expected heterozygosity (gene diversity); *F*_IS_, inbreeding coefficient. (a) With *Carex flava* (sect. *Ceratocystis*, Cyperaceae) and *C. viridula* var. *viridula* from three study regions; (b) *C. flava*, *C. viridula* and hybrid *C.* × *subviridula* from two study regions. Table reports *F* and *P*-values.

(a) *C. flava*, *C. viridula* from three regions													
	df	*N*_e_		Ar		PL %		*H*_o_		*H*_e_		*F*_IS_	
		*F*	*P*	*F*	*P*	*F*	*P*	*F*	*P*	*F*	*P*	*F*	*P*
Taxon	1	3.06	0.09	1.24	0.28	0.13	0.72	7.01	**0.01**	1.54	0.23	1.16	0.29
Region	2	1.56	0.23	2.28	0.12	5.85	**0.01**	0.72	0.50	2.84	0.08	0.06	0.94
Taxa × region	2	2.64	0.09	3.58	**0.04**	5.30	**0.01**	1.94	0.16	3.89	**0.03**	1.35	0.28
(b) *C. flava*, *C. viridula* and *C.* × *subviridula* from two regions													
	df	*N*_e_		Ar		PL %		*H*_o_		*H*_e_		*F*_IS_	
		*F*	*P*	*F*	*P*	*F*	*P*	*F*	*P*	*F*	*P*	*F*	*P*
Taxon	2	2.50	0.10	2.85	0.08	1.19	0.32	6.58	**0.01**	3.34	**0.05**	0.85	0.44
Region	1	2.02	0.17	3.75	0.07	10.2	**0.00**	0.21	0.65	4.55	**0.04**	0.25	0.62
Taxa × region	2	1.39	0.27	0.88	0.43	0.10	0.90	0.30	0.74	0.54	0.59	1.15	0.33

After correcting *P*-values for multiple comparisons, 10 out of 370 tests (10 loci × 37 populations) showed significant deviations from Hardy–Weinberg expectations (Appendix 1). Large positive inbreeding coefficients (*F*_IS_) were generally correlated with significant deviations from Hardy–Weinberg equilibrium (HWE). No locus in any population had a negative inbreeding coefficient that differed significantly from HWE. Locus S180 showed deviations from HWE in three populations (F20, V19, V36), locus Cko2-135 in three populations (F20, F7, V36), locus S177 in two populations (F20, V36) and locus CL101 and locus Cko2-112 in one populations (V36 or F20, respectively). Some pairs (loci × population) could not be tested because loci were not polymorphic.

### Population diversity and inbreeding

The genetic diversity in *C. viridula* populations was highest in Lowland Switzerland (*N*_e_ = 1.77, PL = 70.0 %, Ar = 1.86, *H*_e_ = 0.32), followed by Estonia (*N*_e_ = 1.49, PL = 51.1 %, Ar = 1.59, *H*_e_ = 0.23). In Highland Switzerland only few populations of *C. viridula* were found and their genetic diversity was much lower than in the other two study regions (*N*_e_ = 1.33, PL = 35.0 %, Ar = 1.40, *H*_e_ = 0.16). We expected *C. viridula* with its smaller populations to be more prone to inbreeding. Accordingly, *C. viridula* showed a deficit of heterozygotes in Lowland Switzerland populations and in some populations of Estonia. However, other Estonian populations and the ones in Highland Switzerland showed an excess of heterozygotes (Table 2b).

Mean genetic diversity per population in *C. flava* was highest in Highland Switzerland (*N*_e_ = 1.48, PL = 71.4 %, Ar = 1.67, *H*_e_ = 0.26), intermediate in Lowland Switzerland (*N*_e_ = 1.41, PL = 62.5 %, Ar = 1.55, *H*_e_ = 0.22) and lowest in Estonia (*N*_e_ = 1.37, PL = 37.1 %, Ar = 1.45, *H*_e_ = 0.16). Independent of the region, *C. flava* populations varied in their inbreeding coefficients between −0.79 and 0.65 (Table 2a), the heterozygote excess was not significant, however. These results could be explained first with proximity to several glacial refugia and putative mutagenic influence of higher radiation in mountains.

Genetic diversity was similar for hybrids in Lowland Switzerland (*N*_e_ = 1.49, PL = 65.0 %, Ar = 1.68, *H*_e_ = 0.25) and in Estonia (*N*_e_ = 1.58, PL = 45.0 %, Ar = 1.69, *H*_e_ = 0.24). In both regions hybrid populations showed an excess of heterozygotes (Table 2c), and were more variable than either parental taxon, in line with expectations for populations with genetic admixing.

### Nuclear admixture analysis

**Figure 2.**
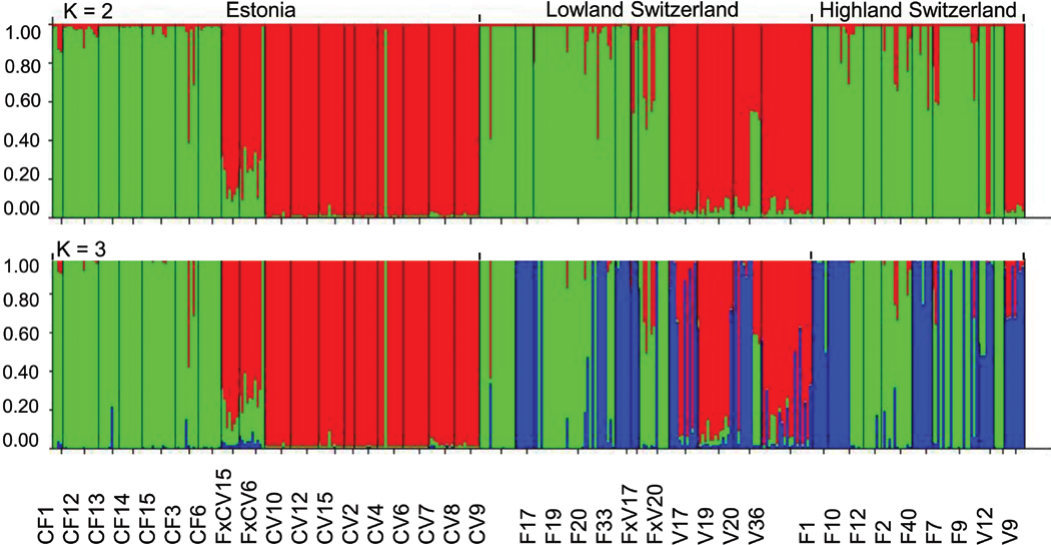
Bar graph illustrating STRUCTURE analysis of *C. flava* (CF/F), *C. viridula* (CV/V) and *C.* × *subviridula* (FxCV/FxV) populations from three studied regions Estonia, Highland and Lowland Switzerland. According to the Δ*K* method, the Bayesian analysis identified two genetic clusters (top, A), while three clusters were found based on observed likelihood values (bottom, B). Each vertical bar represents an individual with coloured partitioning according to genetic clusters. Black vertical lines divide populations. Population names are as in Table 1.

The Δ*K* method in the Bayesian program STRUCTURE classified all individuals into two clusters **[see Supporting Information—Fig. S1]**, illustrating a clear distinction between taxa, but not among regions within the taxa (*K* = 2; Fig. 2A). We used the admixture coefficients from the analysis with *K* = 2 to determine the proportions of admixed individuals. The majority of *C. flava* and *C. viridula* individuals from ‘pure’ populations were indeed classified with high admixture coefficients (*q* > 0.90), i.e. as pure individuals, only two putative *C. flava* and two *C. viridula* individuals had *q* < 0.90, suggesting that they rather were mixed genotypes.

The situation was more complex at ‘mixed’ sites, where two or more taxa of the *C. flava* complex co-occurred. For the *C. flava* morphotypes at the mixed sites 136 individuals of 159 putative *C. flava* were indeed pure *C. flava* (85.5 %; *q* > 0.90), 21 of 159 had mixed genotypes (13.3 %; *q* < 0.90) and two individuals (1.2 %) were even *C. viridula*-like. For the *C. viridula* morphotypes at the mixed sites, 102 individuals of 110 putative *C. viridula* were pure *C. viridula* (92.7 %; *q* > 0.90), three had mixed genotype (2.7 %; *q* < 0.90) and five (4.5 %) were *C. flava*-like. For the *C.* × *subviridula* hybrid morphotypes a wide range of admixture proportions were found (*q* ranged from 0.103 to 0.897), suggesting the presence of a broad range of hybrid generations and backcrossing to both parental taxa.

With the log-likelihood distribution method, the value where the rate of increase in likelihood reaches a plateau without increase in variance corresponds to three clusters **[see Supporting Information—Fig. S1]**. *K* = 3 yielded different results than *K* = 2, also revealing differences between regions, namely admixture between plants of *C. flava* and *C. viridula* in Highland and Lowland Switzerland (Fig. 2B). The third cluster (blue cluster with *q* > 0.1 in Fig. 2B) occurred mostly for individuals at mixed sites of *C. flava* and *C. viridula* (in seven of nine of the mixed sites, and also in Kaltbrunn (F33) and in Melchsee-Frutt (F1), where we exclusively found *C. flava*).

Overall, the STRUCTURE results indicate clear differentiation between the taxa and in addition further variation between regions. This is in line with the results of genetic diversity, which showed significant differences between regions (for the percentage of polymorphic loci) and significant region-by-taxon interactions (for three measures of genetic diversity; Table 3).

### Interspecific differentiation

**Figure 3.**
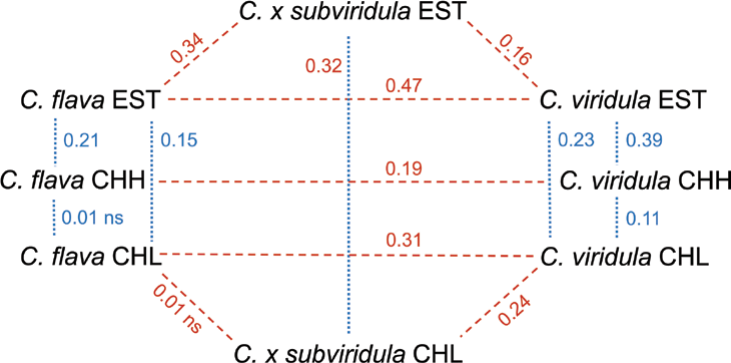
Genetic differentiation (*F*_ST_ values) of *C. flava*, *C. viridula* and *C.* × *subviridula* from three regions of origin, Estonia (EST), Highland (CHH) and Lowland Switzerland (CHL). Dotted blue lines mark intraspecific differentiation between regions, dashed red lines mark interspecific differentiation within regions. Significances of the pairwise *F*_ST_ values were tested using 1000 permutations; all but two comparisons were significant.

**Figure 4.**
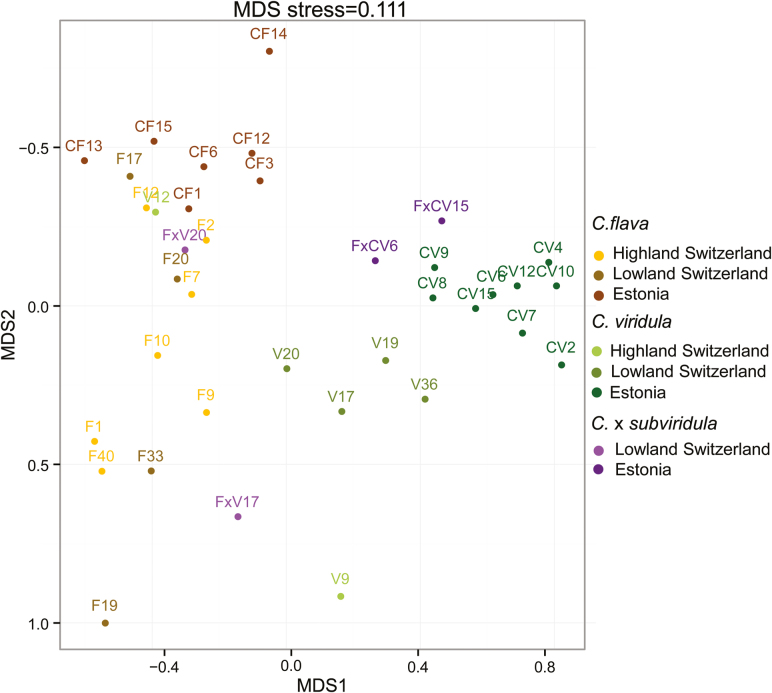
Ordination according to a MDS analysis based on Reynold’s genetic distances between pairs of sampled populations, grouped by the three taxa *C. flava* (CF/F), *C. viridula* (CV/V) and *C.* × *subviridula* (FxV/FxCV) from the three regions Estonia (EST), Highland (CHH) and Lowland Switzerland (CHL). The stress value of 0.11 indicates a good quality of the graphical representation of the MDS analysis. For population codes see Table 1.

According to the hierarchical AMOVA, the proportion of genetic variance within regions between the studied three taxa was highest in Estonia (39.16 %), followed by Highland (28.27 %) and Lowland Switzerland (17.51 %), i.e. between-taxa differentiation was highest in Estonia and lowest in Lowland Switzerland, where three of the five studied sites were mixed (Table 4). AMOVA results corresponded with the *F*_ST_ values between *C. flava* and *C. viridula*, which indicated highest between-taxa differentiation for *C. flava* and *C. viridula* populations in Estonia (*F*_ST_ = 0.47), intermediate in Lowland Switzerland (*F*_ST_ = 0.31) and lowest in Highland Switzerland (*F*_ST_ = 0.19; Fig. 3). In Estonia the hybrid *C.* × *subviridula* was more differentiated from *C. flava* (*F*_ST_ = 0.34) than from *C. viridula* (*F*_ST_ = 0.16), while in Lowland Switzerland the hybrids were more differentiated from *C. viridula* (*F*_ST_ = 0.24) than from *C. flava* (*F*_ST_ = 0.01 ns; Figs 3 and 4).

**Table 4.** AMOVA of *Carex flava* (sect. *Ceratocystis*, Cyperaceae), *C. viridula* var. *viridula* and *C.* × *subviridula* for SSR data considering the whole data set of all 37 populations with two or three hierarchical levels (a and b) or subsets of populations with two or three hierarchical levels (c–i). *P*-value = associated significance derived from 16000 permutations. *P < 0.001; **P < 0.05.

Data set	Source of variation	df	% of variation
(a) 37 populations separated into three groups: Estonia, Highland and Lowland Switzerland	Among groups	2	13.00*
	Among populations	34	33.09*
	Within populations	723	53.90*
(b) 37 populations separated into three groups: *C. flava*, *C. viridula*, *C.* × *subviridula*	Among groups	2	26.56*
	Among populations	34	23.27*
	Within populations	723	50.18*
(c) 33 populations separated into two groups: *C. flava*, *C. viridula*	Among groups	1	32.02*
	Among populations	31	21.20*
	Within populations	663	46.78*
(d) 18 *C. flava* populations grouped by region of origin	Among groups	2	6.84**
	Among populations	15	25.76*
	Within populations	374	67.40*
(e) 15 *C. viridula* populations grouped by region of origin	Among groups	2	20.77*
	Among populations	12	16.07*
	Within populations	289	63.16*
(f) 4 *C*. × *subviridula* populations grouped by region of origin	Among groups	1	**18.27**
	Among populations	2	23.09*
	Within populations	60	58.64*
(g) 18 populations from Estonia grouped by taxa	Among groups	2	39.16*
	Among populations	15	11.01*
	Within populations	316	49.83*
(h) 10 populations from Lowland Switzerland grouped by taxa	Among groups	2	17.51**
	Among populations	7	24.00*
	Within populations	250	58.5*
(i) 9 populations from Highland Switzerland grouped by taxa	Among groups	1	28.27*
	Among populations	7	14.90*
	Within populations	183	56.83*

### Intraspecific differentiation between regions

Intraspecific differentiation between regions was lower for *C. flava* (6.84 %) than for *C. viridula* (20.77 %) or *C.* × *subviridula* (18.27 %) populations (AMOVA, Table 4d–f). In accordance, intraspecific between-region pairwise *F*_ST_ values were slightly lower for *C. flava* (0.01, 0.15 and 0.21) than for *C. viridula* (0.11, 0.23 and 0.39) or *C.* × *subviridula* (0.32; Fig. 3).

Ordination according to the MDS analysis illustrated the genetic distances of populations grouped by taxonomic identity and region of origin (Fig. 4). Estonian *C. flava* populations formed a distinct cluster, whose difference from clusters of Lowland and Highland Switzerland *C. flava* populations was smaller than the differences observed among *C. viridula* populations between the regions (Fig. 4). Estonian *C. viridula* populations clearly differed from Lowland Switzerland populations and even more from the two populations of *C. viridula* in Highland Switzerland (V9 and V12), which were also very different from each other (Fig. 4). These findings clearly suggest higher differentiation in *C. viridula* than in *C. flava*.

Moreover, in combination, the findings on interspecific and intraspecific differentiation indicate that differentiation between the taxa was stronger than differentiation within the taxa between regions. This was further supported by hierarchical AMOVA, where 26.56 % of the variation resided between the three taxa, while 13.00 % resided between regions (Table 4a and b). As expected, the AMOVA analyses without hybrids *C.* × *subviridula* showed higher variance between the parental taxa (32.02 %; Table 4c).

## Discussion

Our microsatellite results support growing evidence that interspecific gene flow is more widespread than previously suspected, but that between-species differences are still retained by various mechanisms (Arnold *et al.* 1990; Friedman *et al.* 2008; Smith and Waterway 2008; Kane *et al.* 2009; Nolte *et al.* 2009; Scascitelli *et al.* 2010). Microsatellite markers are proven appropriate for population structure and differentiation studies (Pálsson 2000; Song *et al.* 2006; Tyagi *et al.* 2016; Volis *et al.* 2016) and for investigating the relationship among closely related taxa (Korpelainen *et al.* 2010; Talve *et al.* 2013, 2014).

We examined whether *C. viridula*, the taxon with a more disjunct distribution and smaller populations, showed lower genetic diversity and higher inbreeding than the more widespread *C. flava*, whose populations are larger and more constant in time. However, mean gene diversity (expected heterozygosity) was slightly higher in *C. viridula*, though not significantly higher from *C. flava* with *H*_e_ = 0.25 and *H*_e_ = 0.21, respectively. *Carex viridula* showed highest genetic diversity in Lowland Switzerland, whereas *C. flava* was most diverse in Highland Switzerland, with *H*_e_ = 0.32 and *H*_e_ = 0.26, respectively. Both taxa had lower diversity in Estonia. Greater allozyme diversity and a lower inbreeding coefficient for *C. viridula* than for *C. flava* was also detected in earlier studies using allozymes by Hedrén (2004) and Bruederle and Jensen (1991), where the latter had considered *C. viridula s.l.*, united with *C. lepidocarpa*, *C. demissa* and *C. viridula s.s.*, however. Meanwhile, Kuchel and Bruederle (2000) detected low levels of allozyme diversity in North American *C. viridula* and attributed it to bottlenecks at arrival from Europe and to predominant selfing. Higher diversity in highland populations, as in our study in *C. flava*, was found in some studies, e.g. for *Cystopteris fragilis* (Pteridophyta) using isozymes (Gämperle and Schneller 2002), for *Primula farinosa* (Primulaceae) using RAPD analysis (Reisch *et al.* 2005) and for *Campanula thyrsoides* (Campanulaceae) (Frei *et al.* 2012), and was suggested to be due to higher mutation rates due to elevated radiation (Li *et al.* 1997). However, others reported higher diversity of lowland populations, as we found for *C. viridula* (e.g. Premoli 2003; Schönswetter *et al.* 2006).

In previous studies on sedges *C. kobomugi* (sect. *Macrocephalae*), *C. macrocephala* (sect. *Macrocephalae*), *C. rugulosa* (sect. *Paludosae*) and *C. scoparia* (sect. *Ovales*), the mean gene diversities of microsatellites were higher (*H*_e_ = 0.589, 0.523, 0.378 and 0.506, respectively) than in our study (*C. flava*, *H*_e_ = 0.21; *C. viridula*, *H*_e_ = 0.25; *C.* × *subviridula*, *H*_e_ = 0.24) (Ohbayashi *et al.* 2008; Hipp *et al.* 2009; King and Roalson 2009; Ohsako 2010). On the other hand, Escudero *et al.* (2010) also detected low levels of gene diversity (*H*_s_ = 0.10) for *C. extensa* (sect. *Spirostachyae*) despite using a wide study area. Possibly our gene diversity values were smaller than the ones detected in other studies, because they generally addressed species with larger and less isolated populations.

Deviation from HWE may indicate inbreeding. In earlier studies, high selfing and evidence for inbreeding has been found in *Carex* (Arens *et al.* 2005; King and Roalson 2009; Escudero *et al.* 2010; Kull and Oja 2010). Inbreeding has also been found to predominate in self-compatible caespitose sedges (Bruederle *et al.* 2008). We found an excess of heterozygotes in *C. flava* when growing adjacent to *C. viridula* (e.g. in populations F12, F19, F17; Table 2a), although this was statistically not significant. The authors who originally published these microsatellites had reported significant excess of heterozygotes in few loci (Ohsako and Yamane 2007; Hipp *et al.* 2009). Earlier it has been shown that *C. flava* is the main partner for backcrossing, as it can occasionally be pollinated successfully by F_1_ hybrids or backcrosses (Schmid 1982; Więcław and Wilhelm 2014). This suggests that *C. flava* was more prone to between-taxa crosses when growing adjacent to other taxa in section *Ceratocystis*, e.g. *C. viridula*, *C. lepidocarpa* and *C. demissa*. On the other hand, the direction of hybridization may be affected by the length of the style (Field *et al.* 2011) which in carices would be determined by the beak length, and hybridization may occur more commonly from long-beaked carices to short-beaked carices due to pollen competition (Derieg *et al.* 2013), which would suggest higher gene flow from *C. flava* to *C. viridula* than vice versa.

We observed hybrids in most of the sites where *C. flava* and *C. viridula* grew together sympatrically. Mean gene diversity (expected heterozygosity) was not higher in hybrid populations than in the parental taxa (*H*_e_ = 0.24 vs. 0.21 and 0.25). Korpelainen *et al.* (2010) studied sedge hybrids in Finland using microsatellite data and found for *C. aquatilis* × *recta* (sect. *Phacocystis*) similar gene diversity than for its parental taxa (*H*_e_ = 0.348 vs. 0.308 and 0.460) and for *C. paleacea* × *recta* higher diversity (*H*_e_ = 0.603 vs. 0.185 and 0.460). In contrast, high genetic diversity, using RAPD analysis, was found in *Fallopia* × *bohemica* (Polygonaceae) in Germany and Switzerland (Krebs *et al.* 2010).

We determined hybrids based on partial sterility and morphological differences from the parental taxa. Most hybrid individuals had utricles without fully developed achenes, but some hybrid individuals had circa 5 % of fully developed achenes (own observation). The admixture proportions detected by microsatellites for hybrid individuals were very variable, indicating that these comprised F_1_ to F_n_ hybrids and backcrosses. Our genetic data showed that some of the supposed intermediate individuals were not hybrids, but rather backcrosses, or in rare cases even pure parental taxa. Thus, our results imply that hybrids can be identified well based on morphological criteria, but that morphological criteria do not allow for distinguishing backcrosses and that they may even lead to occasional, but very rare, errors in identifying pure individuals.

### Interspecific differentiation

The results of our STRUCTURE analysis suggested that we dealt with two species and their hybrids (*K* = 2) and with some regional differentiation (*K* = 3; Fig. 2). The presence of separate taxa was further supported by hierarchical AMOVA, where we detected 13.00 % of genetic variation among studied regions, but more than twice this variation between taxa (32.02 %; Table 4). We conclude that there are solid differences between the two species despite evident hybridization. This is in line with earlier studies on the taxonomic relationships of *C. flava* and *C. viridula* (Bruederle and Jensen 1991; Hedrén 2002; Jiménez-Mejías *et al.* 2012). Similarly, Morgan‐Richards and Wolff (1999) found differences between sympatric *Plantago major* (Plantaginaceae) taxa preserved despite intraspecific gene flow. Kane *et al.* (2009) also concluded that hybridizing *Helianthus* taxa (Asteraceae) remained largely reproductively isolated and morphologically and ecologically distinct despite high levels of interspecific gene flow. Brennan *et al.* (2016) found similar results for hybridizing *Senecio* taxa (Asteraceae) and explained them with selection against hybrids and locally maladapted hybrid individuals.

Our STRUCTURE analysis with *K* = 3 revealed a widely present third genetic cluster in Switzerland, which is extremely rare in Estonia (Fig. 2). This third cluster occurred in all three studied taxa *C. flava*, *C. viridula* and *C.* × *subviridula*, at both low and high altitudes in Switzerland. This suggests high gene flow between taxa within Switzerland and lower between Estonia and Switzerland, as further supported by AMOVA (Table 4). In Estonia, hybridization and introgression occurs, whereas in Lowland Switzerland gene flow between the species seems to be more frequent and to affect the genetic structure of *C. flava* and *C. viridula*.

### Intraspecific differentiation between regions

As expected for rarer taxa with smaller populations, we found higher among-region differentiation for *C. viridula* (AMOVA; 20.77 %) than for the more widespread *C. flava* or hybrid (6.84 and 18.27 %, respectively). In addition, groupwise *F*_ST_ values between regions were higher for *C. viridula*, especially between Estonia and both altitudes in Switzerland (Fig. 3), indicating that hybridization was not strong enough to prevent stronger regional differentiation in *C. viridula*. High levels of differentiation might be caused by low levels of wind pollination, which is not very effective for small herbs in closed habitats (Kull and Oja 2010). Another explanation for the higher differentiation of *C. viridula* is the loss of suitable habitats and subsequent fragmentation of populations, as also shown for other taxa (Pykälä and Toivonen 1994; Reisch *et al.* 2005; Schönswetter *et al.* 2006; Kull and Oja 2010; DeWoody *et al.* 2015). Taking into account that *C. viridula* had a wider distribution in the past and now shows reduced occurrence and increased fragmentation, our finding of higher differentiation between regions for *C. viridula* fits theoretical expectations.

We found especially low genetic differentiation among the *C. flava* groups of populations between Highland and Lowland Switzerland (Fig. 3). This supports the idea of higher gene flow between populations of *C. flava*, as suggested by Schmid (1984b, 1986). With the use of microsatellite markers, Escudero *et al.* (2013) have shown considerable mixing among populations in the widespread *C. scoparia* (sect. *Ovales*) and explained it with long-distance dispersal. Potential for long-distance pollen and seed dispersal was suggested to contribute to low geographic differentiation of circumpolar *C. bigelowii* (sect. *Phacocystis*) (Schönswetter *et al.* 2008). Theoretical studies have shown that only a small amount of long-distance gene flow is needed to prevent population differentiation for neutral alleles (Loveless and Hamrick 1984). Differences in phenology with altitude are expected to reduce gene flow via pollen and increase differentiation instead (Premoli 2003; Reisch *et al.* 2005). As flowering times differ notably in Switzerland (Körner 1999), we suggest that this may explain the observed low differentiation among Swiss *C. flava* populations of similar altitude.

Hybrids showed unexpectedly high differentiation between Estonia and Switzerland (*F*_ST_ = 0.32). This difference could originate from genetic drift in the hybrid populations (Nolte *et al.* 2009). Moreover, backcross patterns may have differed between the regions. A further explanation could be that in natural populations hybrid swarms involve more than two species (Lepais *et al.* 2009). At some sites, we found *C. lepidocarpa* and *C. demissa* growing beside *C. flava* and *C. viridula*, which might increase the number of potential parental taxa and differentiation of hybrids. These mechanisms of hybrid and backcross differentiation between regions are very different from the case of hybrids in the invasive *Fallopia* species complex, which arise from crosses between different taxa in their home origin, and where different hybrids were introduced to different regions, leading to high regional differentiation in the introduced range (Krebs *et al.* 2010).

## Conclusions

Our in-depth analysis of 380 individuals belonging to two sedge taxa and their hybrids and involving populations from three regions suggest that hybridization and introgression are neither strong enough to prevent clear differentiation between taxa nor to prevent stronger regional differentiation for the less common taxon. We encourage further studies on regional differentiation of hybrids and parental taxa to see whether our findings for the *C. flava* complex represent a more general pattern. Moreover, we suggest also considering hybrids and closely related taxa when addressing genetic diversity and differentiation for rare and endangered taxa.

## Sources of Funding

The Estonian Ministry of Education and Research, institutional research funding (IUT 20-28, 20-29) and the European Union through the European Regional Development Fund (Centre of Excellence EcolChange) supported this study.

## Contributions by the Authors

L.S., T.O. and M.F. conceived the study and its design. L.S. conducted field and lab work, analysed data and led the writing of the manuscript. T.O. advised lab work. Both T.O. and M.F. contributed to data analysis, data interpretation and writing of the manuscript. This manuscript forms part of the PhD thesis of the first author (L.S.). All authors have made a substantial contribution to the paper.

## Conflict of Interest

None declared.

## Supporting Information

The following additional information is available in the online version of this article—

**Table S1.** Microsatellite loci used to study genetic diversity and species relationships in *Carex flava* (sect. *Ceratocystis*, Cyperaceae), *C. viridula* var. *viridula* and their hybrid (*C.* × *subviridula*). Presented are primer sequences, 5′ tag, repeat motif, size range, annealing temperature *T*_a_ (°C), number of alleles detected for each locus. The 5′ tags M13R (AGGAAACAGCTATGACCAT) and CAGT (ACAGTCGGGCGTCATCA) were used for incorporation of the fluorescent tag. S082, S180, S245, S175, S119, S177 are from Hipp *et al.* (2009), Cr37 is from Ohbayashi *et al.* (2008), Cko2-112, Cko2-135 are from Ohsako and Yamane (2007), CL101 is from Escudero *et al.* (2010).

**Table S2.** Presence and frequency of private alleles in seven (Cko2-112, Cko2-135, S082, S245, CL101, S180, S175) of the 10 studied microsatellite loci in *Carex flava* (sect. *Ceratocystis*, Cyperaceae), *C. viridula* var. *viridula* and their hybrid (*C.* × *subviridula*). The three other analysed loci (S119, S177, Cr37) did not show private alleles.

**Figure S1.** Estimating the optimal number of clusters with admixture analyses of plants of *Carex flava* (sect. *Ceratocystis*, Cyperaceae), *C. viridula* var. *viridula* and their hybrid (*C.* × *subviridula*) from Estonia, Highland and Lowland Switzerland. (A) The Δ*K* method indicated two genetic groups. (B) The distribution of log-likelihoods indicated three genetic groups.

Supplementry Information
