# Supplementary material for: Two closely related species differ in their regional genetic differentiation despite admixing
Source: AoB Plants. 2018 Jan 29;10(1):ply007. doi: 10.1093/aobpla/ply007 (PMC5817946; doi:10.1093/aobpla/ply007)
Supplement: Supplementry Information [file ply007_suppl_supplementry_information.docx]

Table S1. Microsatellite loci used to study genetic diversity and species relationships in *Carex flava* L. (sect. *Ceratocystis*, *Cyperaceae*), *C. viridula* Michx. var *viridula* and their hybrid (*C.* x *subviridula* Fernald). Presented are primer sequences, 5’ tag, repeat motif, size range, annealing temperature Ta (°C), number of alleles detected for each locus. The 5′ tags M13R (AGGAAACAGCTATGACCAT) and CAGT (ACAGTCGGGCGTCATCA) were used for incorporation of the fluorescent tag. S082, S180, S245, S175, S119, S177 are from Hipp et al. (2009), Cr37 is from Ohbayashi et al. (2008), Cko2-112, Cko2-135 are from Ohsako and Yamane (2007), CL101 is from Escudero et al. (2010).

| Locus | Primer sequence (5`-3`) | 5`-tag | Repeat motif | Size range | T_a_(°C) | alleles |
| --- | --- | --- | --- | --- | --- | --- |
| S082 | F: TGAGAACCCTAGGCAGATGG | CAGT | (GAT)_11_ | 145-170 | 60.1 | 4 |
|  | R: GGGGAAACAAGGTCGTTTAGA |  |  |  |  |  |
| S180 | F: ACATGATTGTGGACGACAGG | CAGT | (GAT)_8_ | 188-191 | 50.6 | 6 |
|  | R: TCACCAAAGTCCTGAAAATCAA |  |  |  |  |  |
| S245 | F: GAAACAAAGGTGCCCCACT | M13 | (CTT)_11_ | 200-236 | 52.4 | 6 |
|  | R: GTTGCAAGCGGGTCTAATTC |  |  |  |  |  |
| S175 | F: TATTGGGTGTGCGATTGAGA | CAGT | (CTT)_8_ | 119-125 | 60.1 | 4 |
|  | R: TCAGATCAGCCAAGTCATCG |  |  |  |  |  |
| S119 | F: CAGTGCTTTTCTGCTTTTCACA | M13 | (CTT)_10_ | 177-189 | 56.4 | 5 |
|  | R: CCACTGCAGCCATTAGTCAA |  |  |  |  |  |
| S177 | F: GCATTGTTTTGTTTGCCTCTT | CAGT | (AAC)_8_ | 171-195 | 49 | 7 |
|  | R: TTCATTTGTGATTGGGTTTCA |  |  |  |  |  |
| Cr37 | F: ACACACACACACAGAGAGAGAG | M13 | (AC)_6_(AG)_5_ | 90-116 | 55.2 | 6 |
|  | R: TTCGATGGAATATCTGCCGTGAG |  |  |  |  |  |
| Cko2-112 | F: CCTTTGCATTATCTCTCTGGAAA | M13 | (CT)_16_ | 220-258 | 55.2 | 11 |
|  | R: AAGCACAAGATCGCAGTTTATGT |  |  |  |  |  |
| Cko2-135 | F: CCTTTACTCCCCATCTCGTCAA | M13 | (AG)_2_GA(AG)_16_ | 208-236 | 49 | 6 |
|  | R: GATGACAACCCACCCCTTGAT |  |  |  |  |  |
| CL101 | F: TTACGAATGTGTGGTGGAATG | CAGT | (CACACAGA)_2_ | 176-194 | 50.6 | 9 |
|  | R: CTTTGTTAGAAGGCATGTACAGTG |  | CACAGA |  |  |  |

Table S2. Presence and frequency of private alleles in seven (Cko2-112, Cko2-135, S082, S245, CL101, S180, S175) of the ten studied microsatellite loci in *Carex flava* L. (sect. *Ceratocystis*, *Cyperaceae*), *C. viridula* Michx. var *viridula* and their hybrid (*C.* x *subviridula* Fernald). The three other analyzed loci (S119, S177, Cr37) did not show private alleles.

| Region and taxa | Locus | Allele | Freq. |
| --- | --- | --- | --- |
| Estonia |  |  |  |
| *C. flava* | Cko2-112 | 244 | 0.015 |
| *C. viridula* | S082 | 170 | 0.012 |
|  | S245 | 212 | 0.083 |
| C. x subviridula | Cko2-112 | 258 | 0.029 |
| Lowland Switzerland | | | |
| *C. flava* | CL101 | 176 | 0.034 |
| *C. viridula* | Cko2-135 | 234 | 0.009 |
| C. x subviridula | - | - | - |
| Highland Switzerland | | | |
| *C. flava* | S180 | 176 | 0.022 |
|  | S175 | 125 | 0.063 |
|  | S175 | 128 | 0.021 |
|  | CL101 | 192 | 0.007 |
|  | Cko2-112 | 232 | 0.014 |
|  | Cko2-112 | 252 | 0.007 |
| *C. viridula* | - | - | - |


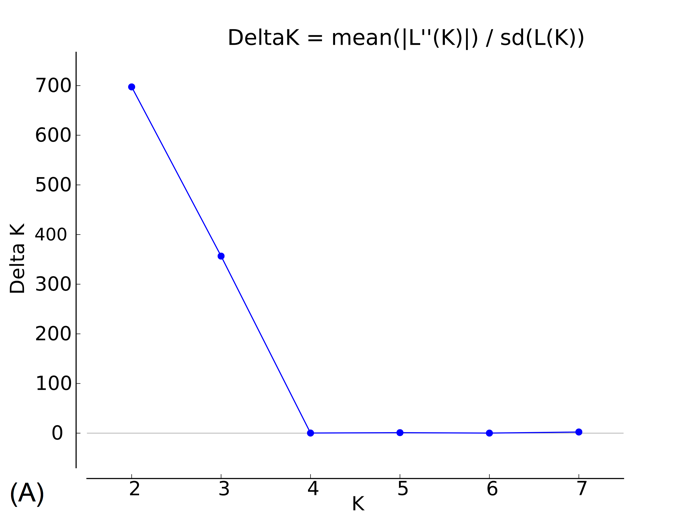

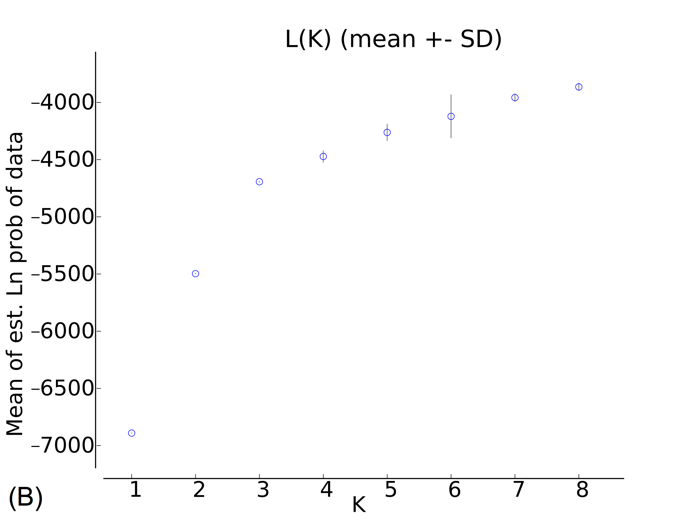


Figure S3. Estimating the optimal number of clusters with admixture analyses of plants of *Carex flava* L. (sect. *Ceratocystis*, *Cyperaceae*), *C. viridula* Michx. var *viridula* and their hybrid (*C.* x *subviridula* Fernald) from Estonia, Highland and Lowland Switzerland. (A) The delta-K method indicated two genetic groups. (B) The distribution of log-likelihoods indicated three genetic groups.
